# The Effectiveness of Cognitive-Behavioral Training on Increasing Self-Concept's Measure and the Attitude Style Toward Narcotic Drugs in Tonekabon Addicted Prisoners

**DOI:** 10.5812/ijhrba.4335

**Published:** 2013-06-26

**Authors:** Ali Reza Mohammadi arya, Mahmoud Shirazi, Abbas Ali Hossien khanzadeh, Fatemeh Lachinnani, Fahimeh Yoosefi Joubari, Zohreh Halajian, Salar Dosti Sarabi, Matloob Ahmed Khan

**Affiliations:** 1 Department Educational Psychology, University of Social Welfare and Rehabilitation Sciences, Tehran, IR Iran; 2 Department of Psychology, University of Sistan and Baluchestan, Zahedan, IR Iran; 3 Department of Psychology, University of Guilan, Rasht, IR Iran; 4 Eighteen District of Ministry of Education, Tehran, IR Iran; 5 Department of Psychology, Islamic Azad University of Tonekabon Branch, Tonekabon, IR Iran; 6 Department of Psychiatry, School of Medicine, University of Addis Ababa, Addis Ababa, Ethiopia

**Keywords:** Cognitive Behavior Therapy, Self Concepts, Attitude, Narcotic Dependence

## Abstract

**Background:**

The existing studies have indicated that persons with positive attitude and believe to narcotics have more addiction than those with negative or natural attitudes. The aim of the present study was to specify the effectiveness of cognitive-behavioral training on increasing of self-concept's measure, and the attitude style toward narcotic drugs in addicted prisoners of Tonekabon.

**Objectives:**

The objective of this study was to assess the effects of cognitive-behavioral training on increasing self-concept and the attitude style toward narcotic drugs.

**Patients and Methods:**

Statistical population included 450 persons. After screening, 65 entered the study. Among them, 40 persons were selected randomly and allocated in experimental and control group. Self-concept and gauge attitude questionnaire were used to gather the data. The program of cognitive–behavioral training was executed at 12 weekly sessions in 120 minutes for experimental group. The past-quest was executed after finishing training for two groups.

**Results:**

The result using analysis of covariance showed that the difference between two groups on self–concept and the attitude toward narcotic drugs was significant at P < 0.5.

**Conclusions:**

Therefore it can be resulted that cognitive–behavioral training is effective on self–concept and the changing of attitude toward narcotic drugs.

## 1. Background

Different researches have indicated the high rate of mental disorders among criminals. Therefore, the aim of jailer is executing correct methods of modification and training to decrease further commission of prisoner’s guilt. So, paying attention toward this issue has been critical by judicial systems in many countries. Totally, the efficiency of cognitive–behavioral therapy has been indicated in decreasing substance abuse and addiction treatment in different researches (1-5). For this reason, we have observed rapid development in cognitive–behavioral intervention programs in prisons and judicial systems in recent years. Therefore we have observed rapid improvement in behavioral–cognitive intervention programs in prisons and judicial systems. Attitude means person's believes about every work resulting, and advocated value for this resulting. Attitudes are logistic reasons of every specific person behaviors. Strong attitudes are the attitudes that we have commitment to them and have extensive reasoning for their accepting (6). The existing studies have indicated that persons with positive attitude have more addiction and believe to narcotics than those with negative or natural attitudes. In this manner, changing natural attitudes to negative is easier than changing positive ones to negative (7). One of the effective factors on humans’ behavior and emotion is the kind of person’s attitude and belief about that accident or in general, that persons scheme about aim matter or phenomenon. This belief has been known as the basic core of cognitive approaches, because cognitive approach believes that the kind of persons belief determines his emotion, behavior and perception, and for this reason, persons believes are the basic aims in cognitive therapy (8). On the other hand, because group therapies are the basic therapy among drug abuse therapies relying upon cognitive methods principle, is one of the most prominent therapies for addicted persons. The therapy which its basic centralization is upon behavior and cognition is called behavioral–cognitive therapy. Behavioral–cognitive therapies need to union and gather between therapies and drug abuser, patient and therapist, survey automatic thoughts irrational believes and patient schemas with substitutes which are not self–destructive. Behavioral–cognitive therapy is based upon life primary experiences or present time, from cognitive style. These cognitive styles can create specified believes which are not applicable to reality (9). According to Rogers in 1951, self-concept is a constructed combination of self-perceptions which is accepted by humans' awareness. This combination is composed of elements like perception, features, personal abilities, and self-concept perception in relationship with others. Persons' attitudes from life realities, morality, and his or her wishes are dependent on the picture that he or she has from himself or herself, i.e. the value which he or she knows for himself or herself. Self-conception which becomes the emotional origin of persons security and if it is threatened, it is like that basic core of self has been threatened (11). Different studies have been performed about behavioral-cognitive therapy in different fields. Ghaharee (1385) showed in a study entitled "The effect of behavioral –cognitive attitude in therapy of Hashish abuse" that behavioral –cognitive attitude has been effective in inclination decreasing, self–possessed increasing, anxiety, and depression decreasing, believes change toward narcotics, and personal abilities increasing emotional intelligence elements (interpersonal abilities, stress bearing compatibility, and general mood) (12). Khodayari Fard *et al.* showed in another study entitled " the survey of effectiveness behavioral –cognitive psycho- analysis, group and personal of prisoners" that the effectiveness of composed and personal therapy is more than control group and psychological interventions, especially group interventions can decrease psychological interventions of prisoners and prevent their return to prison again (13). Shabestan (2010) in a research named "Cognitive–Behavioral Training Effectiveness in the form of groups self-esteem and Attitude of them toward Narcotic Drug in addictions of centers addiction abandonment" showed that training was effective and increased the rate of self-esteem of addictions and created positive changes in their Attitude (14). Aranpoor (1388) showed in one study with this title “the effectiveness of behavioral–cognitive therapy and relaxation training on decreasing of inclination toward cigarette in male students of Yasooj Medical Sciences University" that the attitude of persons who smoke a cigarette and persons who do not smoke is different about narcotics use. i.e. persons who smoke a cigarette have more positive attitude about cigarette smoking than those who do not (15). They also showed that experimental group had more negative attitude than control group toward cigarette smoking after receiving behavioral–cognitive therapy training. The result also showed that experimental group had less inclination towards cigarette smoking showed less abandonment symptoms after training. Literature review shows that there are some studies available about different variables separately. However, we did not find any composed study about the present investigation variables. Therefore, in this research, the effect of behavioral–cognitive therapy on increasing the self–concept and decreasing the positive attitude toward narcotic drugs is studied synchronously.

## 2. Objectives

The objective of this study was to assess the effects of cognitive-behavioral training on increasing self-concept and the attitude style toward narcotic drugs.

## 3. Patients and Methods

This investigation was a pretest-posttest experimental research with control group. 40 male persons seeking help in Tonekabon's prison were selected who had additional 8 months of conviction at the beginning of study. Participants' education level was about guidance school or higher. These participants were selected randomly among males persons seeking help (450 persons) who had above criterion (at least 8 months of conviction remained), and 65 persons who had positive attitude toward narcotic drugs and weak self–concept were screened after performing the attitude–evaluation questionnaire toward narcotic drugs and self- concept questionnaire. Among them 40 persons were selected randomly, and divided into two groups of 20 persons, one experimental group were present at 12 weekly sessions, each session lasting 2 hours of behavioral–cognitive training, and control group were help seekers who were placed in expectation list for receiving training. After finishing training, both groups (control and experimental) answered the questionnaires at the same conditions (situations) again. The condensed content of training was presented at three sessions to control group after performing posttest regarding moral issues.

Attitude-evaluation questionnaire toward narcotic drugs: A and B forms of Nazari questionnaire (1380), for attitude evaluation toward narcotic drugs were used. This questionnaire has been prepared in Likert scale (10). The participants determined their attitude (opinion) as “I agree completely, I agree, I do not have any opinion, I disagree, I disagree completely" for each sentence. Rodgers self- concept questionnaire: self- concept questionnaire by Rodgers has been prepared in 1961. This questionnaire has two forms and was used for measuring the self-concept. Form A includes 25 pair features which was given to the participants. The participants could select from 1 to seven for each pair feature (adjective). After selecting the sample and their relation into experimental and control groups, the questionnaire self-concept and attitude evaluation toward narcotic drugs were performed on experimental and control groups as pretest. Then the programs of behavioral–cognitive training were performed at 12 sessions including 120 minutes on experimental group once a week. After finishing the educational (training) program, posttest was performed for both groups. Finally the date was analyzed using unique factor covariance analysis.

## 4. Results

Unique factor covariance analysis was used for data analysis. The pretest scores of self-concept and posttest scores of attitude toward narcotic drugs as mate variable, and the posttest scores of each of them were analyzed as dependent variable in two separate analyses. The statistical data of unique factor covariance analysis including the natural distribution of scores, the homogeneity of variance, the homogeneity of regression coefficients, and data for divergent quantities were surveyed by graph and also statistical test. The result showed that the homogeneity of variances did not exist in the groups. For this purpose, α = 0.01 was used for testing hypothesis in each analysis. Table 1 shows the mean and standard deviation of pretest and posttest scores for self-concept and attitude toward narcotic drugs on the basis of the groups.

**Table 1.** The Mean and Standard Deviation of the Variable of Self-Concept and Attitude Toward Narcotic Drugs in Experimental and Control Group (Mean ± SD)[Table-fn fn5916]

Group	The Posttest of Positive Attitude Toward Narcotic Drugs	The Pretest of Positive Attitude Toward Narcotic Drugs	Self-concepts Posttest	Self-Concepts Pretest
**Experimental,**	60.80 ± 12.606	80.35 ± 7.995	30.25 ± 6.138	21.65 ± 2.227
**Control,**	80.35 ± 9.906	80.45 ± 9.534	20.65 ± 2.368	20.95 ± 2.523

N = 20, P < 0.1

The first unique factor covariance analysis for predicting self-concepts posttest with group as independent variable and self-concepts pretest as mate variable showed that its effect on self –concept variable is significant (f (1,37) = 66.129; SD = 11.039; P < 0.001; ŋ ^2 ^= 0.647). Persons who received behavioral–cognitive intervention showed more progress from self–concepts pretest to self-concepts posttest than control group (Table 1). This shows that behavioral–cognitive can be effective on the importance of male seekers help and prisons self–concept. The second unique factor covariance analysis for predicting posttest of positive attitude toward narcotic drugs with the group as independent variable and the pretest of positive attitude toward narcotic drugs as mate variable showed that its effect upon the variable of positive attitude toward narcotic drugs is significant.(F (1, 37) = 77.781; SD = 48.623; P < 0.001; ŋ ^2 ^= 0.678). Persons who had received behavioral –cognitive intervention showed more decreasing from the pretest of positive attitude toward narcotic drugs to the posttest of self- concept than control group (Table 1). This shows that behavioral–cognitive intervention can be effective on decreasing positive attitude toward narcotic drugs of male prisoners seekers help.

## 5. Discussion

The aim of the present study was to specify the effectiveness of cognitive–behavioral training on increasing the self- concept and the attitude style toward narcotic drugs in addicted prisons of Tonekabon. The present study indicated that cognitive–behavioral intervention increased prisoners' self-concept, and changed their attitude positively. The result of this study is in accordance with the research results of Irvin (1); Specka et al. (2); McCrady and Ziedonis (3); Maguire et al. (4); Caroll and Onken (5); Ghaharee (12); Aranpour (15); Khodayarifard et al. (13); and Shabestan (14). Also found that after intervention, the prisoners who had participated at group therapy sessions of cognitive–behavioral had better psychological state than the prisoners of control group, and their further conviction has been less. Robinson and Poorporino (11), indicated that cognitive–behavioral interventions of reasoning skill and rehabilitation prevent from renewed return of guilty persons to prison. The finding shows that most prisoners in Tonekabons prison had severe crimes, and then there is the possibility that this intervention for the prisoners with light crimes without any recidivism or less recidivism has higher answering ability. Therefore, it is better to perform this intervention in other prisons about the prisoners with fewer crimes.
